# Aptamer-Based Dual-Cascade Signal Amplification System Lights up G-Quadruplex Dimers for Ultrasensitive Detection of Domoic Acid

**DOI:** 10.3390/md24010050

**Published:** 2026-01-21

**Authors:** Jiansen Li, Zhenfei Xu, Zexuan Zhang, Rui Liu, Yuping Zhu, Xiaoling Lu, Huiying Xu, Xiaoyu Liu, Zhe Ning, Xinyuan Wang, Haobing Yu, Bo Hu

**Affiliations:** 1 Naval Medical Center of PLA, Naval Medical University, Shanghai 200433, China; lijiansengao@163.com (J.L.); ey_zhangzexuan@163.com (Z.Z.); 13855723380@163.com (R.L.); ningzhe95@163.com (Z.N.); xywang0521@163.com (X.W.); 2 Lab of Biosystem and Microanalysis, State Key Laboratory of Bioreactor Engineering, East China University of Science and Technology, Shanghai 200237, China; zhenfeixu321@163.com (Z.X.); xuhuiying@ecust.edu.cn (H.X.); 3 College of Marine Food and Bioengineering, Jiangsu Ocean University, Lianyungang 222005, China; 4 College of Basic Medical Sciences, Naval Medical University, Shanghai 200433, China; zhuyuping72@hotmail.com (Y.Z.); luxiaoling80@126.com (X.L.)

**Keywords:** domoic acid, aptasensor, G-quadruplex dimer, signal amplification, food safety

## Abstract

In recent years, harmful algal blooms have led to frequent occurrences of shellfish toxin contamination, posing a significant threat to the safety of aquatic products and public health. As a potent neurotoxin, domoic acid (DA) can accumulate in shellfish, highlighting the urgent need for rapid and highly sensitive detection methods. In this study, we developed a fluorescent aptasensor based on a dual-signal amplification system by combining G-quadruplex (G4) dimers with multi-walled carbon nanotubes (CNTs). The sensor is designed with a hairpin-structured aptamer as the recognition probe, where short multi-walled CNTs serve as both a fluorescence quencher and platform, and G4 dimers are incorporated into the sensing interface to enhance signal output. In the absence of the target, the hairpin-structured aptamer remains closed, keeping the fluorescence signal “off”. Upon binding to DA, the aptamer undergoes a specific conformational change that exposes the G4-dimer sequence. The exposed sequence then binds to thioflavin T (ThT), which in turn generates a greatly enhanced fluorescence signal, leading to a substantial fluorescence enhancement and completing the second stage of the cascade amplification. Under optimal conditions, the constructed sensor achieves rapid detection of DA within 5 min, with a low detection limit of 1.1 ng/mL. This work presents a valuable tool for the rapid and sensitive detection of DA in shellfish, with promising applications in marine environmental monitoring and food safety regulation.

## 1. Introduction

Domoic acid (DA) is a potent neurotoxin produced by marine diatoms such as Pseudo-nitzschia [1,2,3]. Due to its structural mimicry of glutamate, DA binds with high affinity to ionotropic glutamate receptors in the central nervous system, thereby inducing amnesic shellfish poisoning [4]. Beyond gastrointestinal symptoms like vomiting and diarrhea, DA exerts profound neurotoxicity, leading to confusion and short-term memory loss [5,6]. In severe cases, it may cause permanent neurological damage or even fatalities [7,8,9]. With the increasing frequency of harmful algal blooms in coastal waters, DA contamination has intensified [2]. This neurotoxin accumulates significantly in shellfish through the food chain, presenting a persistent and serious threat to global seafood safety and public health.

Currently, the routine detection of DA primarily relies on instrumental and immunological methods [5,10], such as high-performance liquid chromatography–mass spectrometry (HPLC-MS), enzyme-linked immunosorbent assay (ELISA), lateral flow assays, and various immunosensor platforms [8,9,11,12]. Although HPLC-MS is considered the “gold standard” for confirmatory analysis, its cumbersome procedures and reliance on sophisticated instrumentation hinder its application in rapid testing [13,14]. While ELISA provides greater operational convenience, its dependence on costly antibodies, batch-to-batch variability, and susceptibility to matrix effects restricts its widespread adoption and reliability [15,16,17]. Recently, biosensing platforms employing nucleic acid aptamers have garnered increasing interest for detecting marine neurotoxins such as DA [18,19]. Several DA-specific aptamers have been developed, exhibiting dissociation constants typically in the µM range, which inherently limits sensitivity for direct detection [20,21,22]. While signal amplification strategies applied to other toxins (e.g., okadaic acid, saxitoxin) have achieved lower detection limits, they often rely on complex, multi-step operations [23,24,25,26]. To enable simple yet sensitive DA detection, we designed a novel chimeric aptamer. It conjugates a high-affinity DA-binding sequence with a G4 dimer, creating an integrated recognition and amplification module within a single DNA strand.

To address the core challenges of signal sensitivity and interference resistance, signal amplification strategies have become pivotal [27]. Among these, the specific binding system between G-quadruplex (G4) and thioflavin T (ThT) has been widely adopted for constructing label-free biosensors [28], owing to its high signal-to-noise ratio and unique conformational switching properties [29,30,31]. However, the conventional G4/ThT system suffers from a fluorescence intensity that is susceptible to the concentration of monovalent cations in the medium, along with an inherently limited signal output [32]. Consequently, it poses a significant challenge for the direct ultrasensitive detection of trace toxins in complex samples. Notably, G4 dimers, higher-order structures formed by guanine-rich sequences, can bind ThT within a more stable spatial configuration [33,34], yielding a substantially enhanced fluorescence signal [35]. This discovery offers a promising avenue for developing advanced sensing platforms.

**Figure 1:**
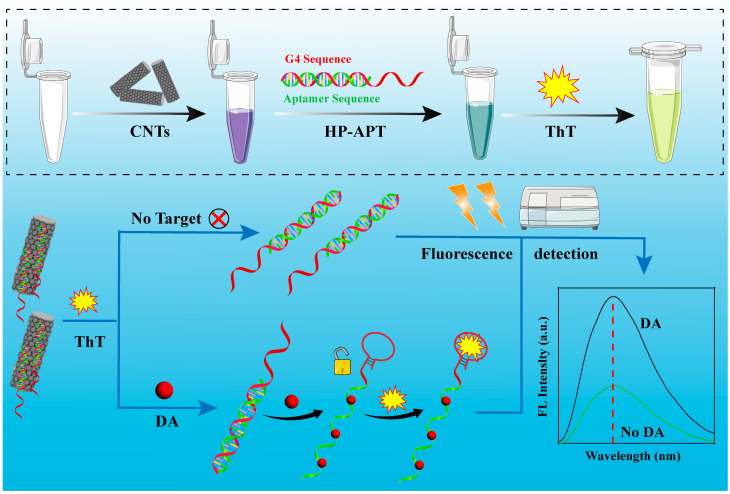
Schematic illustration of the aptasensor fabrication and its working mechanism for DA detection.

Based on this, we designed a novel dual-cascade signal amplification system for the ultrasensitive detection of DA. This aptasensor employs an integrated two-stage amplification system comprising initial background suppression and subsequent signal enhancement. The initial “signal-on” stage utilizes the exceptional quenching efficiency of short multi-walled carbon nanotubes (CNTs) and competitive aptamer dissociation to drastically suppress the background. Subsequently, target-induced unfolding of a hairpin aptamer (HP-APT) probe releases a G4 dimer, which binds ThT to yield a markedly stronger fluorescence signal than a G4 monomer, constituting the second amplification stage (Figure 1). The synergy between “quenching-release” and “G4 dimer enhancement” in this dual-cascade design marries high specificity with high sensitivity, enabling ultrasensitive detection within minutes and presenting a robust solution for rapid DA detection.

## 2. Results

### 2.1. Characterization of Aptasensor

**Figure 2:**
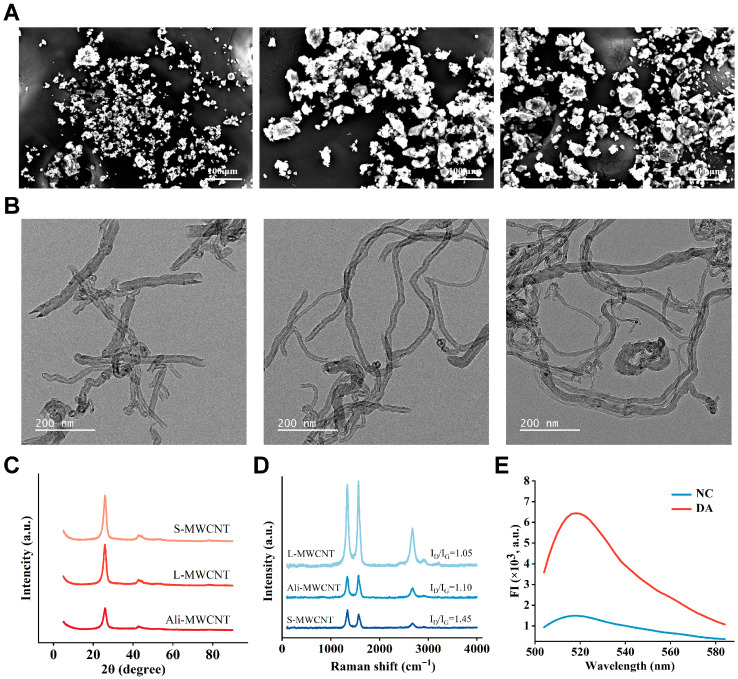
Characterization of the sensor: (**A**) SEM images of different carbon nanotubes: short multi-walled CNTs, long multi-walled CNTs, and aligned multi-walled CNTs. Scale bar: 100 μm. (**B**) TEM images of different carbon nanotubes: short multi-walled CNTs, long multi-walled CNTs, and aligned multi-walled CNTs. Scale bar: 200 nm. (**C**) XRD pattern of the CNTs. (**D**) Raman spectroscopy of the CNTs. (**E**) Fluorescence intensity map verifying the feasibility of the sensing mechanism.

As shown in Figure 2A, scanning electron microscopy (SEM) analysis of the three CNT types revealed distinct morphological features: short multi-walled CNTs exhibited uniform dispersion and a narrow length distribution, conducive to stable suspension; long multi-walled CNTs spanning tens of micrometers showed significant entanglement; and aligned multi-walled CNTs formed ordered bundles. As shown in Figure 2B, transmission electron microscopy (TEM) provided clear morphological visualization of the three types of MWCNTs, directly confirming the structural differences described above. Short multi-walled CNTs, benefiting from their reduced dimensions and superior dispersibility, offer a larger specific surface area and a more uniform quenching interface. Therefore, they are crucial for achieving more homogeneous and efficient fluorescence quenching [36,37]. X-ray diffraction (XRD) analysis reveals that all three carbon nanotube samples exhibit a characteristic (002) diffraction peak around 26°, corresponding to the graphitic interlayer spacing (Figure 2C). The crystallite sizes along the c-axis, calculated using the Scherrer equation from the full width at half maximum (FWHM) of the (002) peak, decreased in the order: L-MWCNTs (97 nm) > Ali-MWCNTs (82 nm) > S-MWCNTs (64 nm). This trend quantitatively corroborates the variation in structural order among the samples. Notably, the short multi-walled CNTs show a peak with a slightly larger full width at half maximum. Raman spectroscopy (Figure 2D) revealed distinct D and G bands at approximately 1350 cm^−1^ and 1580 cm^−1^, respectively, in all analyzed samples. Among them, the short multi-walled carbon nanotubes exhibited the highest ID/IG intensity ratio, indicating a greater density of structural defects. While such defects may marginally compromise electrical conductivity, they can significantly enhance fluorescence quenching efficiency via π-π stacking and non-radiative energy transfer mechanisms [38,39]. Furthermore, a moderate level of defects increases the number of surface-active sites, thereby facilitating the adsorption and interaction of the aptamers [40]. As shown in Figure 2E, the presence of DA triggers a specific conformational change in the aptamer probe upon binding, which releases the G4 dimer sequence. The liberated G4 dimers then bind to ThT, resulting in a substantial fluorescence recovery. This effective “signal-off to signal-on” transition demonstrates the feasibility of the designed sensing mechanism.

### 2.2. Optimization of G4 Dimer Sequences

**Figure 3:**
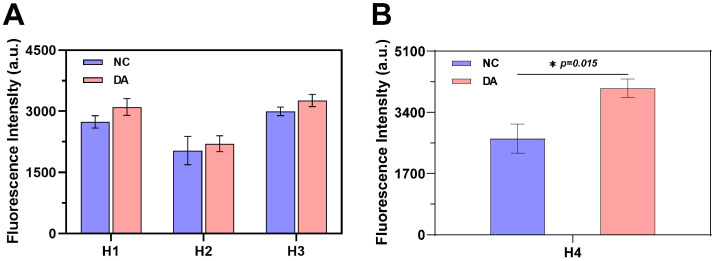
Comparative fluorescence analysis of G4 dimer hairpin sequences: (**A**) The initial G4 dimer hairpin sequence (H1–H3) (n = 3). (**B**) The optimized H4 hairpin sequence (n = 3).

To identify optimal G4 dimers for DA sensing, we constructed three hairpin probes (H 1–H 3) by fusing a DA-specific aptamer with literature-sourced G4 dimer sequences (Table 1) [21,22,23]. The fluorescence intensity at 485 nm was determined using a microplate reader after co-incubation of the annealed probes with ThT and DA. As shown in Figure 3A, probes H 1 and H 3 display comparable fluorescence intensities, which are substantially greater than that of H 2. Notably, none of the probes exhibited significant fluorescence changes upon DA addition, indicating an impaired conformational transition. We attribute this to the excessive length and high stability of the stem region, which likely prevented the structural reorganization required for signal activation. Given its compact structure and shorter sequence length, we selected the H 3 G4 dimer as the template for all further probe optimization.

To improve the response performance, probe H 4 was engineered from H 3 by shortening the stem region to reduce the number of complementary base pairs. As shown in Figure 3B, the structural optimization of the H 4 probe enabled a significant DA-induced fluorescence enhancement. We attribute this signal turn-on to a two-step mechanism: first, DA binding triggers the dissociation of the hairpin structure, exposing the G4 dimer; subsequently, the freed G4 dimer specifically binds to ThT, yielding the amplified fluorescence signal.

### 2.3. Optimization of Experimental Conditions

**Figure 4:**
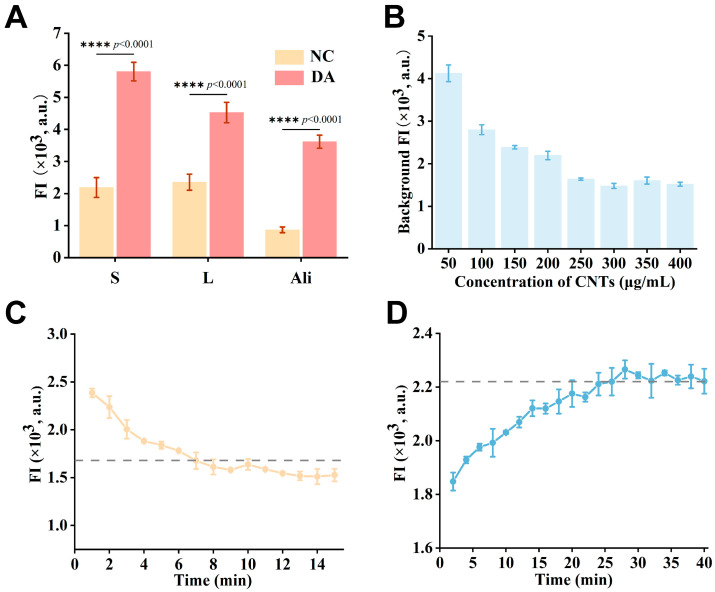
Optimization of key parameters for the G4/CNT-based aptasensor: (**A**) Fluorescence quenching efficiency of different types of CNTs (n = 3). (**B**) Fluorescence intensity of the sensor at varying CNT concentrations (n = 3). (**C**) Effect of fluorescence quenching incubation time on the background signal (n = 3). (**D**) Effect of fluorescence recovery incubation time on the detection signal (n = 3).

To achieve optimal detection performance of the constructed sensor, we conducted a systematic optimization of four key parameters: the type and concentration of CNTs, fluorescence quenching time, and fluorescence recovery time. The selection of CNTs, which function as both fluorescence quenchers and adsorption substrates, was a critical determinant of aptamer loading and subsequent ThT signal generation. Figure 4A demonstrates that although all three CNTs achieved fluorescence quenching, their efficiency and functional utility were distinctly governed by their specific properties. Despite yielding a greater normalized response (≈260% vs. ≈160% for short multi-walled CNTs), the aligned multi-walled CNTs produced a lower absolute fluorescence signal after DA addition. The superior absolute signal strength of the short multi-walled CNT system provides a wider detection dynamic range, which was therefore selected for all subsequent experiments. This can be attributed primarily to the larger specific surface area and the high density of accessible binding sites provided by the fragmented, short nanotubes, which collectively enhance the adsorption of ThT and thereby significantly improve the photoinduced electron transfer efficiency. Critically, this structural configuration achieves efficient fluorescence quenching while preserving the conformational flexibility essential for the adapter’s function. Therefore, in the presence of the target, it enables more effective probe dissociation and subsequent signal recovery. This distinct advantage thus established short multi-walled CNTs as the optimal platform for all subsequent experiments. To determine the optimal concentration, we evaluated the effect of short multi-walled CNT concentration on the ThT background fluorescence. As shown in Figure 4B, the background signal decreased progressively with increasing CNT concentration and stabilized at 250 μg/mL, which was therefore selected as the optimal working concentration.

The fluorescence quenching time determines the establishment of adsorption equilibrium between ThT and the CNT surface, which directly influences the stability of the background signal [41]. Kinetic analysis (Figure 4C) reveals that fluorescence intensity drops rapidly and stabilizes within approximately 8 min, marking the completion of the quenching equilibrium. Extending the incubation time to 15 min did not yield a significant increase in fluorescence intensity. Considering the practical need for rapid detection, an incubation time of 8 min was therefore determined to be optimal. The fluorescence recovery kinetics, reflecting the dynamic process of DA binding to its aptamer, constitute a critical determinant of detection sensitivity. As shown in Figure 4D, a rapid increase in fluorescence intensity occurred within the first 10 min after DA addition, followed by stabilization at approximately 25 min. The fluorescence signal plateaued after 25 min of incubation, with no further increase upon extended reaction times. Thus, 25 min was selected as the optimal condition to ensure a strong signal while maintaining a fast assay workflow.

### 2.4. Analytical Performance of the Sensor

**Figure 5:**
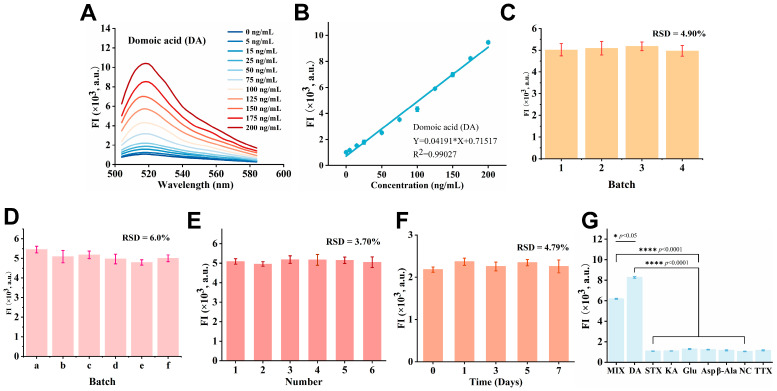
Analytical performance of the DA fluorescence aptasensor: (**A**) Fluorescence emission spectra of the sensor upon addition of DA at varying concentrations. (**B**) Corresponding calibration curve showing the linear relationship between fluorescence intensity and DA concentration (n = 3). (**C**) Reproducibility across different sensor batches (n = 3). (**D**) Reproducibility of sensors from the same batch (n = 3). (**E**) Repeatability of the sensor response across six independently prepared assays (n = 3). (**F**) Long-term stability of the sensor signal stored at 4 °C over a period of 7 days (n = 3). (**G**) Specificity test against common interfering marine toxins and amino acids (n = 3).

To systematically evaluate the analytical performance of our sensor, we assayed its response to DA across a range of concentrations. As shown in Figure 5A, the fluorescence intensity increased regularly with DA concentration, thus demonstrating a positive dose–response relationship. To enable precise quantification, we plotted a standard curve with DA concentration (ng/mL) on the *x*-axis and corresponding fluorescence intensity values on the *y*-axis. As shown in Figure 5B, fluorescence intensity exhibited a good linear relationship with DA concentration across the range of 5.0 to 200 ng/mL. The corresponding standard curve is fitted to the equation Y = 0.04191X + 0.71517, with a linear correlation coefficient (R^2^) of 0.99027. Based on the detection limit calculation formula, the sensor’s detection limit was determined to be 1.1 ng/mL. This ultra-low value is well below the statutory limit for DA in shellfish (20 μg/g), providing sufficient early warning capability for safety regulation [22,23]. Moreover, our method demonstrates superior sensitivity compared to standard ELISA and offers a significantly faster and more cost-effective alternative to HPLC (Table 2). A key feature of this detection strategy is its dual-signal enhancement mechanism, which synergistically combines the strong quenching of short multi-walled CNTs with the conformational change-driven signal amplification of the aptamer. This design enables ultrasensitive DA detection without relying on enzymatic reactions or nanomaterial labeling, offering a straightforward and robust approach.

### 2.5. Specificity and Stability of the Sensor

The repeatability and stability of a detection method are critical for assessing its practical utility [43]. To evaluate repeatability, six parallel measurements of a DA standard were conducted under identical conditions. Furthermore, intra-batch reproducibility was evaluated using a larger batch of six sensors, showing an RSD of 6.0%. Inter-batch reproducibility (RSD = 4.9%) was also confirmed (Figure 5C,D). As shown in Figure 5E, the relative standard deviation (RSD) for six parallel measurements was 3.70%, which is below the conventional acceptance threshold of 5%, indicating excellent repeatability and precision of the method for DA detection. To evaluate the storage stability, sensors from the same batch were stored at 4 °C in the dark and tested with an identical DA concentration at 0, 1, 3, 5, and 7 days. The 7-day stability test yielded an RSD of 4.79% for the signal (Figure 5F). These results demonstrate that the sensor exhibited robust stability under appropriate storage conditions, supporting its viability for practical applications.

Specificity, a crucial performance metric for aptasensors, dictates their utility in complex samples [44]. We therefore evaluated the method’s specificity for DA by testing cross-reactivity with key interferents: the marine neurotoxins saxitoxin (STX) and kainic acid (KA); and the structurally analogous amino acids glutamic acid (Glu), aspartic acid (Asp), tetrodotoxin (TTX), and β-alanine (β-Ala). As shown in Figure 5G, the groups containing DA (either alone or in mixture) exhibited a strong fluorescence signal approximately 8–10 times higher than the blank control. In contrast, all single interfering substances yielded signals indistinguishable from the blank control.

### 2.6. Real-Life Sample Analysis

To validate the reliability of this method for practical application, we performed spiked recovery experiments. Water samples were spiked with DA standard solutions at low, medium, and high concentrations, with three replicates at each level. As shown in Table 3, the average recovery rates for DA ranged from 90% to 102%, with RSD consistently below 6%. This demonstrates that the developed sensing platform possesses good accuracy and precision, thereby meeting the requirements for practical sample analysis.

## 3. Discussion

In summary, we successfully developed a highly sensitive and rapid fluorescence aptasensor for DA detection, leveraging a dual-cascade signal amplification system based on G4 dimers and short multi-walled CNTs. This sensor achieves superior detection performance by leveraging the high quenching efficiency of short multi-walled CNTs and a dual-cascade process within the G4 dimer/ThT system, which integrates target-induced conformation change with subsequent signal amplification. The first stage exploits the superior quenching capability of short multi-walled CNTs to minimize background fluorescence. Upon competitive binding of DA, the aptamer is released from the CNT surface, initiating the first signal transduction step via the restoration of the fluorescence signal. In the second stage, the liberated aptamer sequence folds into a stable G4-dimer, which binds ThT with high specificity, generating a dramatically enhanced fluorescence signal compared to the monomeric form and thus achieving the second stage of amplification. The developed fluorescent aptasensor enables rapid detection of DA within 33 min, with a notably low detection limit of 1.1 ng/mL. The aptasensor exhibited satisfactory accuracy in actual sample testing, with recovery rates ranging from 90.7% to 102.3%. These results validate its reliability and hold promise for its application in marine environmental monitoring and food safety.

## 4. Materials and Methods

### 4.1. Materials and Apparatus

L-Alanine (L-Ala), saxitoxin (STX), and kainic acid (KA) were obtained from Meryer (Shanghai, China). Domoic acid (DA) and Thioflavin T (ThT) were purchased from Sigma-Aldrich (Louis, MO, USA). Aspartic acid (Asp), tetrodotoxin (TTX), and β-alanine (β-Ala) were purchased from Aladdinn (Shanghai, China). Glutamate (Glu) was purchased from Heowns Biochem Technologies LLC (Tianjin, China). All reaction buffers were prepared in phosphate-buffered saline (PBS) and diluted to the specified concentrations (10 mM, pH 7.4) with ultrapure water. The morphology was characterized using a transmission electron microscope (Tecnai G2F20, FEI, Hillsboro, OR, USA) and a scanning electron microscope (Hitachi S-3400N, Tokyo, Japan). The fluorescence curves were recorded on a fluorescence spectrophotometer (Hitachi, F-2500, Tokyo, Japan).

### 4.2. Fluorescence Detection DA

First, 2.0 mg of short multi-walled carbon nanotubes was dispersed in 1.0 mL of ultrapure water. Then, the mixture was sonicated in an ice bath for 10 min to obtain a stable dispersion. For the quenching assay, 88 µL of assay buffer, 15 µL of the CNT dispersion, and 2 µL of 10 µM ThT were sequentially added to a microplate well. Subsequently, 5 µL of 1 µM HP-APT was added, making a total volume of 110 µL. The mixture was gently mixed and incubated at 25 °C to allow the aptamer to adsorb onto the CNT surface and quench the ThT fluorescence.

Following the quenching incubation, 10 µL of a standard or sample solution containing DA was added to each well. After gentle mixing, the mixture was incubated at 25 °C for 25 min to allow DA to competitively displace the aptamer from the CNT surface, resulting in fluorescence recovery. The fluorescence intensity was then measured.

### 4.3. Calculation of the Detection Limit (LOD)

The detection limit for DA based on the IUPAC definition (signal-to-noise ratio S/N = 3) was calculated using the linear function and the following equation:

LOD = 3σ/S, where σ is the standard deviation of the blank solution. S is the slope of the linear calibration plot between the fluorescence emission intensity and the concentration of DA.

### 4.4. Specificity

The specificity was investigated using common marine toxins (STX, TTX, and KA) and structurally similar amino acids (Glu, Asp, β-Ala, etc.) as potential interfering agents. These substances were introduced at concentrations equivalent to that of DA to conduct the interference tests.

## Figures and Tables

**Table 1:** Sequences of the G4 dimer-hairpin probes designed for DA detection.

Name	Sequence (5′-3′)
H 1	**G GGT AGG GCG GGT TGG GGG GTA GGG CGG GTT GGG **CA CGG GTT GGG AAC *GAG GTG TGT ACA CCG TG* CCC AAC
H 2	**GGGG TAG GGC GGG TTG GG **CA CGG TT GGG AAC *GAG GTG TGT ACA CCG TG* CCC AA
H 3	**GGG TTA GGG TTA GGG TTA GGG** CA CGG TTA GGG AAC *GAG GTG TGT ACA CCG TG* CCC TAA
H 4	**GGG TTA GGG TTA GGG TTA GGG **CA CGA ATA GGG AAGGACA *GAG GTG TGT ACA CCG TG* CCC TAA

Note: The underlined sequence represents the complementary sequence of the hairpin loop, the bold sequence denotes the G4 dimer sequence, and the italicized sequence indicates the DA aptamer sequence.

**Table 2:** Comparison of the sensor and other reported sensors for DA determination.

Analytical Method	Materials	Linear Ranges	LOD	Detection Time	Reference
Molecularly imprinted polymer	NanoMIPs	0.011–1000 nM	4.32 nM	45 min	[6]
Electrochemical	PDA-rGO/PAM	1–600 nM	0.31 nM	8 min	[7]
HPLC	Apt@ZIF-8	20–200 ng/mL	7.0 ng/mL	15 min	[21]
Biolayer interferometry	Aptamer	0.625–10 μM	13.7 nM	7 min	[24]
Lateral flow immunoassay	Antibody	1.4–60 ng/mL	1.4 ng/mL	30 min	[42]
Fluorescent	Aptamer	20–200 ng/mL	1.1 ng/mL	5 min	This work

**Table 3:** Recovery results of DA in spiked samples.

Analytical Method	Added (ng/mL)	Measured (ng/mL)	Recovery (%)	RSD (%)
Fluorescent aptasensor	10	9.1	90.7	5.6
	50	51.8	98.3	2.1
	100	100.8	102.3	3.3

## Data Availability

The original contributions presented in this study are included in the article. Further inquiries can be directed to the corresponding authors.

## Abbreviations

The following abbreviations are used in this manuscript:

DA	Domoic acid
G4	G-quadruplex
CNTs	Carbon nanotubes
ThT	Thioflavin T
HPLC-MS	High-performance liquid chromatography–mass spectrometry
ELISA	Enzyme-linked immunosorbent assay
HP-APT	Hairpin aptamer
L-Ala	L-Alanine
STX	Saxitoxin
KA	Kainic acid
AA	Ascorbic acid
GSH	Glutathione
Cys	Cysteine
SEM	Scanning electron microscopy
XRD	X-ray diffraction
RSD	Relative standard deviation
